# Severity of imposter syndrome associated with resilience, self-esteem, and depression among medical students in Thailand

**DOI:** 10.3389/fpubh.2025.1577184

**Published:** 2025-09-05

**Authors:** Arpunna Suriyasathaporn, Vithawat Surawattanasakul, Nuntaporn Karawekpanyawong, Ranlaphat Aungkasuraphan, Disatorn Dejvajara, Wuttipat Kiratipaisarl

**Affiliations:** ^1^Faculty of Medicine, Chiang Mai University, Chiang Mai, Thailand; ^2^Department of Community Medicine, Faculty of Medicine, Chiang Mai University, Chiang Mai, Thailand; ^3^Environmental Medicine and Occupational Medicine Excellent Center, Faculty of Medicine, Chiang Mai University, Chiang Mai, Thailand; ^4^Department of Psychiatry, Faculty of Medicine, Chiang Mai University, Chiang Mai, Thailand

**Keywords:** imposter syndrome, resilience, self-esteem, depression, medical student

## Abstract

**Objective:**

This study aimed to determine the prevalence of Imposter Syndrome (IP) and its associated factors among Thai medical students.

**Methods:**

Thai medical students voluntarily participated in this cross-sectional survey conducted from September to October 2022. The completed online questionnaires included the Clance Imposter Phenomenon Scale (CIPS) to assess IP Status, along with several other factors from different questionnaires: the Single-Item Measurement of Suicidal Behaviors, the Patient Health Questionnaire, the Resilience Inventory, and the Rosenberg Self-Esteem Scale. Linear regression analyses were conducted to examine the associations between these factors and IP.

**Results:**

A study involving 477 medical students revealed that 47.1% of Thai participants reported experiencing frequent IP, while 7.1% identified at an intense level. Notably, students in their 2nd year, along with those exhibiting high resilience, demonstrated lower CIPS scores when compared to their 1st-year counterparts, with regression coefficients of −4.72 and −9.66, respectively. Conversely, factors such as moderate and high self-esteem, as well as indicators of depression, were significantly associated with an increased severity of IP.

**Conclusion:**

Approximately 50% of Thai medical students experience Impostor Syndrome, which is associated with higher rates of depression. Additionally, high self-esteem may worsen IP. Promoting resilience through structured curricula and group therapy overseen by medical schools could be an effective approach to address this issue.

## 1 Introduction

Impostor Syndrome (IP), also known as the impostor phenomenon, is a psychological experience characterized by persistent self-doubt despite objective successes and a fear of being discovered as a fraud. IP is commonly observed among medical students. Additionally, organizational culture plays a significant role in the emergence of IP, and the prevailing culture in medicine, which fosters a fear of failure, can be detrimental (1, 2). IP can have various implications for medical education, potentially leading to differences in learning styles. This highlights the need to tailor curricula to accommodate the considerable number of learners experiencing IP.

The prevalence of IP varies across different professions, with rates ranging from 23.7 to 50.0%. Graduate students experience IP rates between 25.8 to 33.3% (3–5), while college students report rates between 40.0 and 50.0% (6, 7). Medical students experience IP prevalence from 23.7 to 49.4% (8–10), and other professions have rates between 24.0 to 39.0% (11–14). Factors contributing to these variations include internal elements like self-evaluation and self-perception (15), and external influences such as family dynamics and workplace culture (16). IP can lead to physical symptoms, including headaches, stomach aches, and insomnia, as well as mental health issues such as psychological distress, low self-esteem, depression, and burnout (8, 17, 18).

The IP can lead to significant consequences such as anxiety, self-criticism (19, 20), and even suicidal ideation in severe cases (15, 21). In medical schools, IP is associated with decreased performance, poor professional identity, and lack of confidence, all of which increase stress in a competitive environment (22, 23). To cope, processes like self-reflection and self-evaluation can foster resilience and self-confidence (24). Effective strategies include reflective journaling, structured supervision, and group workshops, which can reduce scores on the Clance Impostor Phenomenon Scale (CIPS) and enhance self-awareness and self-confidence (25).

Despite the notable prevalence of IP among medical students, there is a significant gap in research concerning Thai medical colleges. This study aims to assess the prevalence of IP among this population and identify the factors associated with it.

## 2 Materials and methods

### 2.1 Participants, study design, and data collection

This study was conducted as a cross-sectional survey involving Thai medical students from their first to sixth year, between September and October 2022. Data were collected through a secure website accessible only to authorized personnel. Participants volunteered after being informed via Facebook (Overcoming Imposter Project), Instagram (imposter.syndrome.bootcamp2022), and a Line^®^ application group, the most popular instant messaging and voice-over-IP service in Thailand. In collaboration with the Society of Medical Students of Thailand, letters were also sent to Thai medical institutions to inform them about the study. Participants provided informed consent before completing Thai-language questionnaires anonymously, which included their region, year of study, and academic performance. The survey assessed factors such as the CIPS, the Single-Item Measurement of Suicidal Behaviors, the Patient Health Questionnaire (PHQ-9) for depression, the Resilience Inventory (RI-9), and the Rosenberg Self-Esteem Scale (RSES).

Upon completing the survey, participants received automatically generated results for the CIPS, the Single-Item Measurement of Suicidal Behaviors, PHQ-9, RI-9, and RSES, along with individualized feedback for each measure provided by a psychiatrist. They were also given contact information for free psychological services available to all Thai medical students, including a website, hotline, and Facebook page for the Department of Mental Health. The study’s sample size was calculated using Biostatistics, yielding a total of 384 participants, based on parameters *d* = 0.05, *p* = 0.474, and alpha = 0.05, from a cross-sectional study on the Impostor Phenomenon among medical students in Thailand (26). Participants needed to be medical students in their 1st to 6th years in 2022, have internet access, and be able to read Thai, while those studying outside Thailand were excluded. Eligibility was confirmed through two questions: “Are you a 1st to 6th year medical student?” and “Are you a medical student studying in Thailand?” Only those who answered “Yes” to both could proceed with the survey.

### 2.2 Study variables

The prevalence of IP among the students, as the outcome variable, was determined using the CIPS, originally developed by Clance and Imes (27) and validated in Thai by Chaisaen (18). The CIPS consists of 20 items rated on a six-point scale (0 = strongly disagree to 5 = strongly agree), with the total score ranging from 0 to 100. Scores were categorized into four levels of IP: mild (0–40), moderate (41–60), frequent (61–80), and intense (81–100) (28). A score of 61 or higher indicates that the individual has experienced IP (28). The overall Cronbach’s alpha for this scale was 0.96, which indicated good internal consistency (29).

The study identified factors affecting IP severity as indicated by CIPS scores, including student region, age, medical year, and GPA, as well as suicidal ideation, depressive disorder, resilience, and self-esteem.

Data from the Single-Item Measurement of Suicidal Behaviors were utilized to evaluate participants’ experiences of suicidal feelings over the past year (30). Participants indicated “yes” or “no” to whether they had experienced suicidal thoughts in the last 12 months. This measurement indicated a false positive rate of 10.7% and a false negative rate of 6.39% (31).

The depressive status was assessed using the PHQ-9, a validated nine-item tool for screening and monitoring major depressive disorder (MDD). Participants rated their symptoms on a 4-point scale from 0 (“Not at all”) to 3 (“Almost every day”), with total scores ranging from 0 to 27. A score of 9 or higher indicated potential major depression, with a sensitivity of 0.84 and specificity of 0.77 compared to the Thai Mini International Neuropsychiatric Interview (32). Depression severity was categorized as mild (9–14 points), moderate (15–19 points), and severe (≥20 points) (33).

The RI-9, a validated nine-item instrument, was used to assess an individual’s ability to cope with adversity. Participants responded on a 5-point Likert scale, where 1 point means “does not describe me at all” and 5 points mean “describes me very well.” Total scores range from 9 to 45. Participants were categorized based on their scores: below the 25th percentile reflects low resilience, between the 25th and 75th percentile indicates average resilience, and above the 75th percentile indicated high resilience (34). The instrument has a strong reliability with a Cronbach’s alpha of 0.90 (35).

The RSES is a validated 10-item instrument designed to measure an individual’s self-worth and self-esteem. Participants rated their level of agreement with each statement using a 4-point Likert scale: 1 point for “strongly disagree,” 2 points for “disagree,” 3 points for “agree,” and 4 points for “strongly agree.” Scores ranged from 10 to 40, with the following interpretations: low self-esteem (10–25), medium self-esteem (26–29), and high self-esteem (30–40) (36). The Cronbach’s alpha for the Thai version of the RSES was found to be 0.86 (37), and this version was utilized in the present study.

The study followed the Declaration of Helsinki, and the protocol received approval from the Research Ethics Committee of the Faculty of Medicine at Chiang Mai University, Thailand (Study code: FAC-MED-2565-09115).

### 2.3 Statistical analysis

Data are presented as counts and percentages for categorical variables, and as means and standard deviations or medians and interquartile ranges (IQR) for continuous variables, depending on normality. Normality was assessed using Q–Q plots and histograms. The prevalence of IP is calculated as the number of individuals with IP in each category divided by the total number of participants. The numerical CIPS score from each student served as the outcome variable to evaluate IP severity in simple linear regression models, which examined factors like student demographics, suicidal ideation, depressive disorders, resilience, and self-esteem. Each academic year was treated separately as a categorical variable due to its unique context. Established cut points for the RSES, RI-9, and PHQ-9 were also used. The regression coefficients indicated the relationship between IP severity and associated factors.

The study findings adhered to the Strengthening the Reporting of Observational Studies in Epidemiology statement guidelines (38). After data collection, the missingness patterns were reviewed. Observations with completely at random missing data (MCAR) were excluded, and a frequency-based inverse probability weighting was used to account for missing data at random (MAR) from the sampling variability across pre-clinical and clinical years. For missing data not at random (MNAR), we did not perform imputation. A sensitivity analysis was conducted using the difference in beta (dfbeta) method to assess influential observations and result robustness. Robust variance correction was applied for highly influential observations, and all analyses were conducted using STATA version 16.0.

## 3 Results

### 3.1 Data description

**Figure 1:**
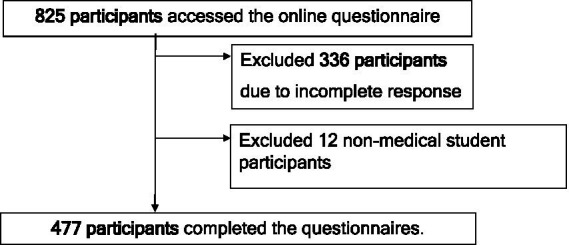
Study flow diagram demonstrating the recruitment process in the 1st – 6th year medical students across Thailand from September to October 2022.

The study flow diagram of participants is shown in Figure 1. A total of 477 completed all questionnaires. The distribution of respondents is as follows: North (57.2%), Bangkok (18.0%), Northeast (15.7%), Central-West–East (7.3%), and South (1.6%). The demographic characteristics of the participants are summarized in Table 1. The mean ± SD age of the participants was 21.0 ± 2.0 years, with a minimum age of 18 years and a maximum age of 44 years. Most participants (76.5%) were in their preclinical years (1st–3rd years), with approximately 20% in each of these years. The number of respondents in the clinical years (4th–6th year students) was lower than in the preclinical years. Based on the multiple-choice questions with a 0.5-grade interval of academic grades, over half (54.1%) of the participants reported high academic performance scores (3.51–4.00), while the 25th percentile fell between 3.01 and 3.5. The scores for resilience, self-esteem, and depression were reported as medians of 33 (IQR 29-37), 26 (IQR 25-27), and 8 (IQR 5-13), respectively. Many participants reported moderate levels of resilience and self-esteem. Interestingly, almost half reported experiencing depression within the past 2 weeks, and one-fifth indicated having thoughts of suicide. Data was initially assumed to be MNAR in the first section, which included age and academic year. After the CIPS section, the data was considered MCAR due to a loss of 2% (9 out of 486) across the sections of the questionnaire, which comprised the RSES, RI-9, PHQ-9, and measures of suicidal ideation. As a result, no imputation for missing data imputation was performed.

**Table 1:** Descriptive baseline and sociodemographic characteristics of participating 1st – 6th year medical students across Thailand (September to October 2022).

Baseline characteristics	*n* (%)
Age (median, IQR)	21.0 (20.0, 22.0)
Academic year	
Students in the 1st year	104 (21.8)
Students in the 2nd year	134 (28.1)
Students in the 3rd year	127 (26.6)
Students in the 4th year	47 (9.9)
Students in the 5th year	28 (5.9)
Students in the 6th year	37 (7.8)
Grade point average^a^	
0.00–2.50	39 (8.2)
2.51–3.50	180 (37.7)
3.51–4.00	258 (54.1)
Resilience^b^	
Low	101 (21.2)
Moderate	259 (54.3)
High	117 (24.5)
Self-esteem^c^	
Low	169 (35.4)
Moderate	268 (56.2)
High	40 (8.4)
Depression^d^	211 (44.2)
Suicidal ideation^e^	101 (21.2)

^a^Indicates grade point average; the lowest grade point average is 0, the highest is 4. A higher-grade point average means a higher academic performance.

^b^Indicates Resilience as determined by the Resilience Inventory (RI-9). A score below the 25th percentile (0–28 points) indicates low resilience, between the 25th and 75th percentiles (29–37 points) indicate average resilience, above the 75th percentile (38–50) indicates high resilience.

^c^Indicates Self-esteem as determined by the Rosenberg Self-Esteem Scale (RSES). A score of 0–10 indicates low Self-Esteem, 11–20 indicate moderate, 21–40 indicate high.

^d^Indicates Depression as determined by the Patient Health Questionnaire (PHQ-9). A score of less than 9 indicates the absence of depression, 9 or more indicates the presence of depression.

^e^Indicates Suicidal ideation as determined by the Single-Item Measurement of Suicidal Behaviors (SIMSB). A score of 0 indicates the absence of suicidal ideation, 1 indicates the presence of suicidal ideation.

### 3.2 Imposter syndrome

The overall mean and SD of the CIPS score were 58.3 and 16.0, respectively. As shown in Table 2, 40% of Thai medical students reported frequently experiencing IP, while only 7.1% reported intense IP.

**Table 2:** Mean (standard deviation) and frequency and proportion across severity categories of imposter syndrome scores in the 1st – 6th year medical students across Thailand (September to October 2022).

Score of imposter syndrome	Mean (SD)	*n* (%)
Few (0–40)	18.6 (12.2)	66 (13.8%)
Moderate (41–60)	51.1 (6.0)	186 (39.0%)
Frequent (61–80)	69.3 (5.7)	191 (40.0%)
Intense (81–100)	86.3 (4.3)	34 (7.1%)

### 3.3 Factors associated with imposter syndrome

The results from exploratory multivariable linear regression models, which included robust standard error correction, are presented in Table 3. Students in their 2nd exhibited lower levels of IP severity compared to 1st-year students, with CIPS regression coefficients of −4.72 (95% CI: −7.97, −1.47). No differences were found among students in other academic years. Additionally, higher levels of resilience were associated with less severe IP. Students with high resilience showed a notably greater decrease in severity, with a regression coefficient of −9.66 (95% CI: −15.40, −3.91) compared to those with low resilience.

**Table 3:** Multivariable linear regression demonstrating factors associated with imposter syndrome, using CIPS scores, the dependent variable, in the 1st – 6th year medical students across Thailand (September to October 2022).

Determinants	Beta-coefficient (95%CI)
Academic year	
Students in the 1st year	(reference category)
Students in the 2nd year	−4.72 (−7.97, −1.47)
Students in the 3rd year	−2.64 (−6.34, 1.07)
Students in the 4th year	1.91 (−4.23, 8.06)
Students in the 5th year	0.53 (−7.12, 8.18)
Students in the 6th year	1.08 (−5.78, 7.93)
Age (years), per 1-year increase	−0.44 (−1.49, 0.60)
Grade point average^a^	
0.00–2.50	−4.77 (−10.86, 1.32)
2.51–3.50	(reference category)
3.51–4.00	−0.18 (−3.92, 3.56)
Resilience^b^	
Low	(reference category)
Moderate	−2.75 (−7.16, 1.66)
High	−9.66 (−15.40, −3.91)
Self-Esteem^c^	
Low	(reference category)
Moderate	4.41 (0.83, 7.98)
High	16.26 (11.03, 21.48)
Depression^d^	10.19 (6.21, 14.16)
Suicidal ideation^e^	3.43 (−0,96, 7.83)
Constant	64.51 (43.3, 85.72)

^a^The lowest grade point average is 0, the highest is 4. A higher-grade point average means a higher academic performance.

^b^Resilience was determined by the Resilience Inventory (RI-9). A score below the 25th percentile (0–28 points) indicates low resilience, between the 25th and 75th percentiles (29–37 points) indicate average resilience, above the 75th percentile (38–50) indicates high resilience.

^c^Self-esteem was determined by the Rosenberg Self-Esteem Scale (RSES). A score of 0–10 indicates low Self-Esteem, 11–20 indicates moderate, 21–40 indicates high.

^d^Depression was determined by the Patient Health Questionnaire (PHQ-9). A score of less than 9 indicates the absence of depression, 9 or more indicates the presence of depression.

^e^Suicidal ideation was determined by the Single-Item Measurement of Suicidal Behaviors (SIMSB). A score of 0 indicates the absence of suicidal ideation, 1 indicates the presence of suicidal ideation.

Higher self-esteem and depression were linked to an increase in the severity of IP. Students with moderate self-esteem experienced a 4.41 regression coefficient (95% CI: 0.83, 7.98) increase in severity compared to those with low self-esteem. In contrast, students with high self-esteem showed a significant regression coefficient increase of 16.26 (95% CI: 11.03, 21.48) in severity. Additionally, students reporting depression had a 10.19 regression coefficient (95% CI: 6.21, 14.16) increase.

## 4 Discussion

### 4.1 Prevalence

This study found that 42.8% of Thai medical students surveyed experienced IP using a cutoff CIPS score of 61. The prevalence of IP among medical students worldwide ranges from 30.6 to 87% (22, 39–41). For example, 30.6% of individuals are affected in Peru, and 45.2% in the Middle East (22). In the USA, prevalence rates range from 58 to 87%, while in Canada, it is reported at 73% (42). Although IP has not been widely reported in Asia, our findings suggest that Thai medical students also experience it. The prevalence among these students is similar to that in South America and the Middle East, though it may be lower than in higher-income countries like the USA and Canada. Most studies on IP have been conducted outside of Asia. One Asian study linked high parental expectations, common in many Asian families, to IP. However, the prevalence we found is not excessively high, indicating that other factors may reduce imposter feelings. Future research should explore this with a larger sample size and a wider range of factors.

### 4.2 Factors associated with the severity of imposter syndrome

#### 4.2.1 Demographics

This study found that 2nd^-^year medical students had a CIPS regression coefficient decrease of −4.72 (95% CI: −7.97, −1.47), compared to 1st-year students. A study from the United Kingdom (43) indicates that feelings of impostor syndrome often intensify during critical transitional life phases, such as the transition to university. Therefore, the score reduction observed in the 2nd-year could be attributed to the relatively stable environment of preclinical years, which minimized the need for constant adaptation and, in turn, resulted in lowered impostor syndrome scores. However, the prevalence of impostor syndrome among medical students remains controversial across various social contexts. Studies conducted in Brazil (44) and Ireland (22) have shown no significant differences in the prevalence of impostor syndrome among students across different academic years. Conversely, a study conducted in the USA (39) reported a significant increase in impostor scores during the final year compared to the first year when students entered university. Unlike findings related to the academic year, our study found that age differences did not affect the severity of IP. Other studies have also reported no differences in the severity of IP between age groups (22, 45, 46).

#### 4.2.2 Psychological issues

This study found that depression were linked to increased IP severity, with CIPS regression coefficients of 10.19. IP. Researchers have also established positive connections between IP and depression in Peruvian medical students (41), Thai medical students (26), and Egyptian nursing students (47). Experiencing stress and being surrounded by more successful peers can intensify these feelings (41, 48). While IP is not a mental illness, it can contribute to mental health issues by increasing stress and negative self-perception. Conversely, depression may also evoke feelings of inadequacy similar to IP. Recognizing the bidirectional relationship between IP and depression is essential, as they can reinforce each other.

#### 4.2.3 Self-esteem

According to Table 3, 1st to 6th years students with high self-esteem in this study exhibited higher scores for IP. This is the first report of the positive relationship between high self-esteem and more severe IP in Thailand. In contrast, most previous studies involving medical students in other regions have found a relationship between low self-esteem and IP (22, 40, 41, 48). Differences in the population’s culture, lifestyle, and mental health may explain this finding. Additionally, Asian individuals with lower self-esteem often adopt an emotionally driven coping style to manage stressors, and lower self-esteem is associated with reduced anxiety in the Asian population (49).

While high self-esteem and IP may initially appear to be contradictory (22, 40, 41, 48, 50), they can coexist. In fact, high self-esteem can sometimes lead to IP due to the pressure of high expectations (51), competition (17, 52), fear of judgment (53, 54), and the need to maintain a positive self-image. The occurrence of high expectations starts before entering medical school, with impressive academic records and achievements that significantly contribute to their acceptance. As a result, they tend to set very high standards for themselves (55). They encounter substantial academic and professional pressures due to the demanding medical curriculum, exposure to extensive and complex information, and constant evaluations by professors, preceptors, and peers. This pressure can lead to feelings of inadequacy, as students may feel overwhelmed and doubt their ability to master all aspects of their studies. Such feelings can threaten their self-image and self-worth (56–58). In the medical school environment, students find themselves surrounded by exceptionally motivated and talented peers (59). Individuals with high self-esteem often engage in a cycle of self-comparison with their classmates, which can lead to feelings of inadequacy when they perceive others excelling. This ongoing self-evaluation can intensify the impact of IP. Students with high self-esteem may feel increased anxiety about receiving negative feedback (60, 61). This anxiety can worsen IP when they receive criticism or constructive input (53, 62), making them question the legitimacy of their admission and attributing their success to luck or external factors rather than recognizing their abilities. In addition, individuals with high self-esteem tend to have a fear of judgment and often strongly desire to maintain a positive self-image (63). They may worry that admitting gaps in their knowledge or seeking help could diminish how competent their peers perceive them to be. This fear of potential judgment can trigger IP when they face challenges or make mistakes (17).

#### 4.2.4 Resilience

Our results indicate that higher resilience was associated with lower CIPS scores. Participants with high resilience experienced a significant reduction in IP severity, with a CIPS regression coefficient of −9.66. Similarly, a study conducted in Saudi Arabia identified a negative correlation between two factors among undergraduate nursing and medical students, evidenced by a significant correlation coefficient of r = −0.220 (64). This correlation was also observed in a study among Brazilian medical students (65). Resilience, the ability to adapt to stress, serves as a protective factor against IP, leading to fewer depressive symptoms and burnout (65).

### 4.3 Strengths and limitations

This study is one of the few to investigate the impact of IP on Thai medical students, revealing both negative factors like depression as well as positive aspects such as resilience. These findings could inform positive psychology policies for medical students. However, this study has some limitations. First, there are challenges in measuring IP prevalence through online surveys, as satisfied individuals may opt out. Statistical methods, sample size calculation, and an approach to informing students on a variety of platforms were used to address imbalances and enhance the integrity of our findings. A more standardized sampling approach could be implemented to improve sample representation. Second, the cross-sectional nature of the study limits the ability to establish causal relationships. Most parameters related to behavior were presented some time before the study was performed. However, the time occurrence of some parameters and outcomes is uncertain. Therefore, the result of our study must be carefully applied, and adopting longitudinal designs in future studies could clarify the relationship. Finally, as the questionnaire-based survey cannot collect qualitative information, future research should consider exploring alternative methods, such as interviews or focus groups, that could provide more insight into the field.

### 4.4 Practice implications and intervention suggestions for medical schools

This study found that high self-esteem correlates with high IP, indicating that even confident, high-performing students can experience self-doubt. Therefore, educators should not assume students are immune to psychological struggles. Medical schools should routinely assess students’ mental health, provide accurate and constructive feedback, normalize conversations about mental health, offer support, and integrate resilience-building programs into the curriculum, as these have been shown to enhance resilience (66, 67).

## 5 Conclusion

Almost half of Thai medical students face IP, similar to medical students in other countries. This condition is linked to higher risks of other mental health issues, including depression. This study found that individuals with higher self-esteem may also experience the impostor phenomenon. Future studies should implement a more standardized sampling approach to improve representation and adopt longitudinal designs to clarify the relationship. Medical educators and schools should regularly assess students for stress and insecurity, monitor their psychological health, provide follow-up care for those showing signs of depression, and integrate resilience-building programs into the curriculum to better support them.

## Data Availability

The raw data supporting the conclusions of this article will be made available by the authors, without undue reservation.
